# Supplementary figures and images for: Characterising Complex Enzyme Reaction Data
Source: PLoS One. 2016 Feb 3;11(2):e0147952. doi: 10.1371/journal.pone.0147952 (PMC4740462; doi:10.1371/journal.pone.0147952)

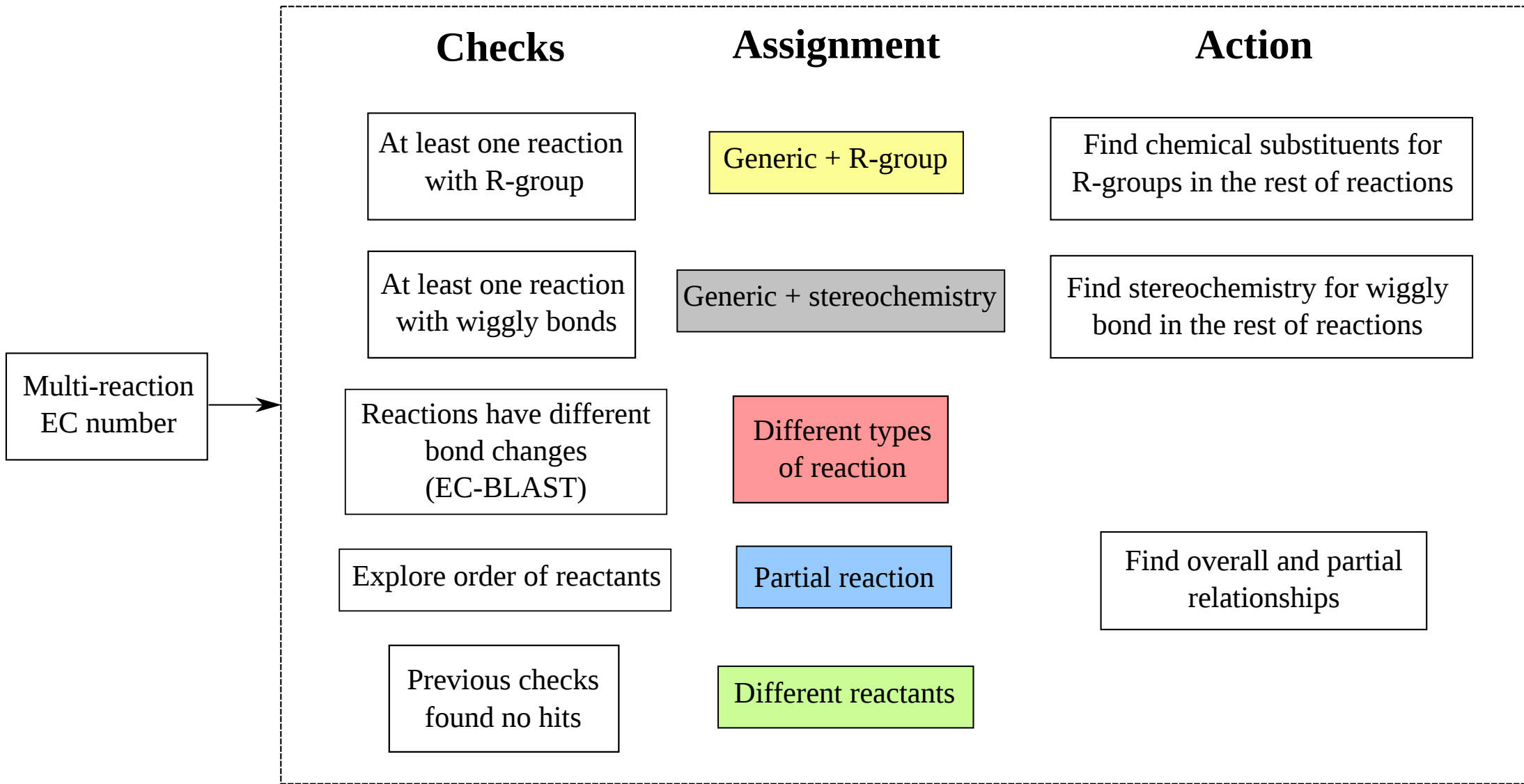

Supplement: S1 Fig — (PDF) [file pone.0147952.s001.pdf]

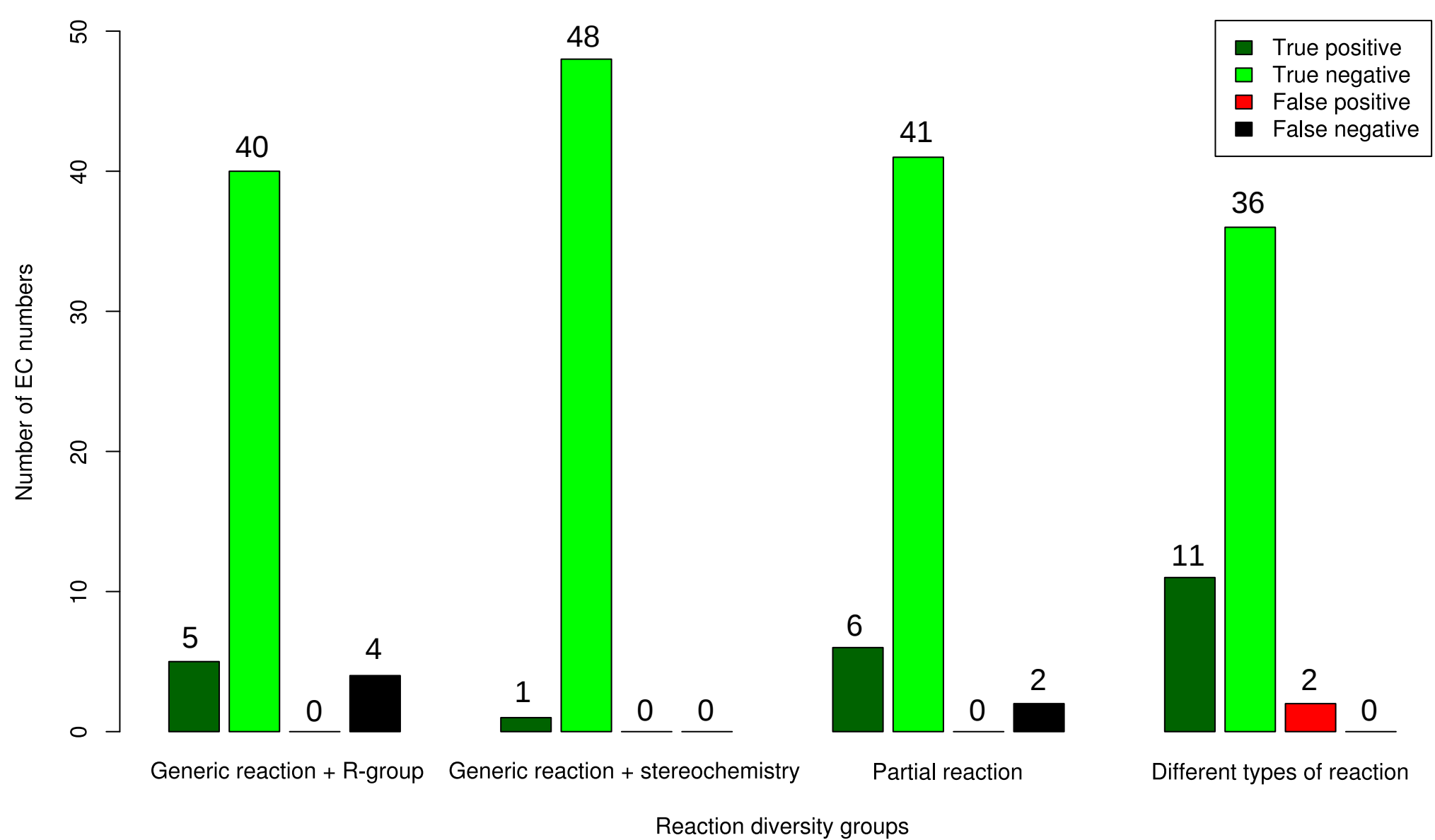

Supplement: S2 Fig — (PDF) [file pone.0147952.s002.pdf]

## 2-Acetolactate mutase (EC 5.4.99.3)

### *Generic reaction - stereochemistry*

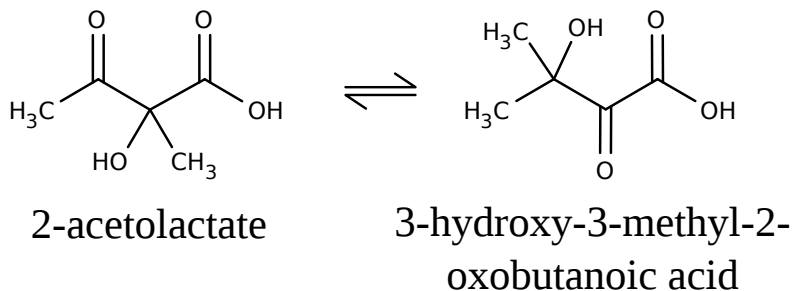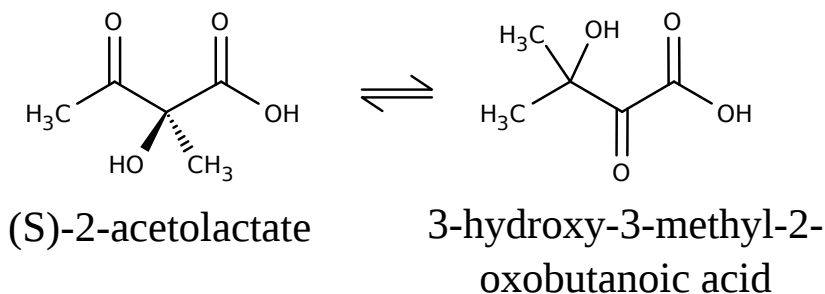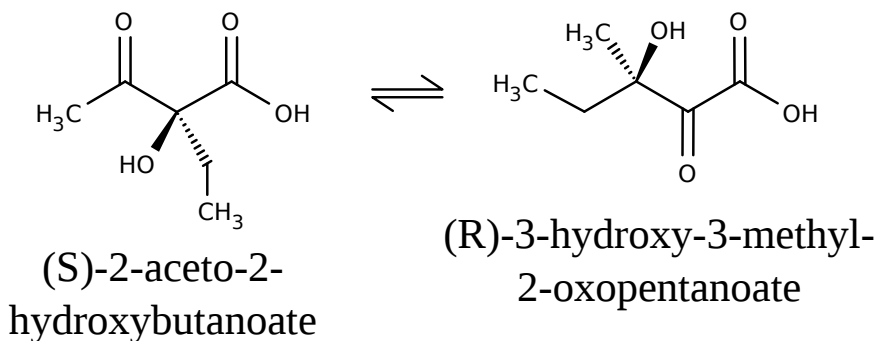

*Different reactants*

Supplement: S3 Fig — (PDF) [file pone.0147952.s003.pdf]
