# Supplementary material for: Characterising Complex Enzyme Reaction Data
Source: PLoS One. 2016 Feb 3;11(2):e0147952. doi: 10.1371/journal.pone.0147952 (PMC4740462; doi:10.1371/journal.pone.0147952)
Supplement: S4 Fig — (a) Enoate Reductase (EC 1.3.1.31) is an oxidoreductase acting on different reactants. The type of the reaction, the bond changes in both reactions are the same whereas the reactants are different. (b) Phenol Beta-Glucosyltransferase (EC 2.4.1.35) is an example of generic reaction on the basis of R-group. The common scaffold is black and the variable chemical substituent is highlighted in green. (c) Glucose-1-Phosphatase (EC 3.1.3.10) is an example of generic reaction based on stereochemistry. The stereochemistry of glucose is represented with wiggly bond (undefined) or down (defined) bond and highlighted in green. (d) Trypanothione Synthase (EC 6.3.1.9) catalyses two subsequent reactions leading to trypanothione production from glutathione and spermidine. (i) glutathionylspermidine production from glutathione and spermidine using ATP, (ii) trypanothione production from glutathione and glutathionylspermidine using ATP. Intermediate compound (glutathionylspermidine) is highlighted in green. (e) Acetylenedicarboxylate decarboxylase (EC 4.1.1.78) catalyse different types of reactions. This EC also exemplifies partial chemistry as the overall process involves hydration of 2-Hydroxyethylenedicarboxylate and decarboxylation of acetylenedicarboxylate formed by the first reaction to produce pyruvate. (PDF) [file pone.0147952.s004.pdf]

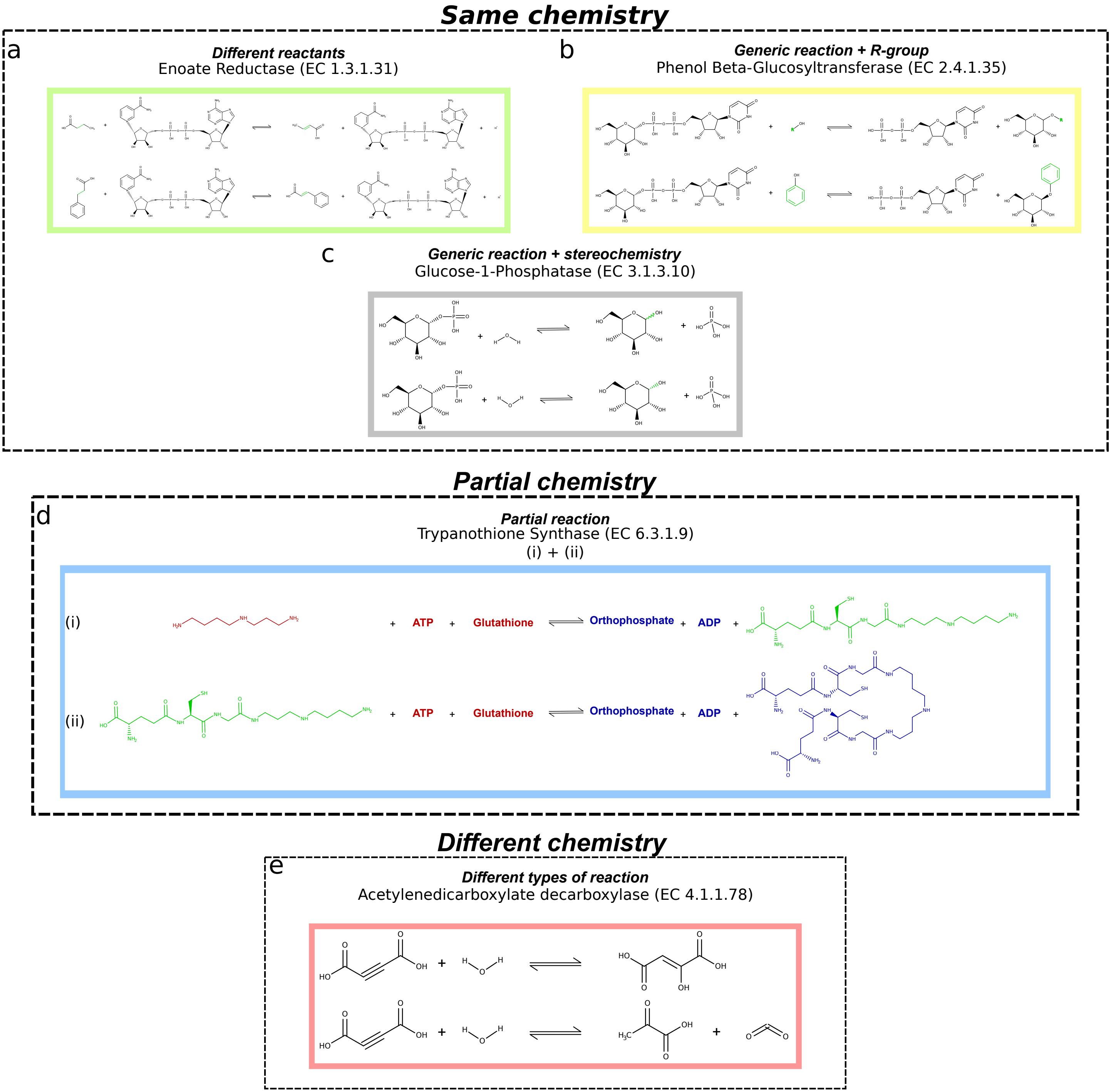
Same chemistry
a
Different reactants
Enoate Reductase (EC 1.3.1.31)
b
Generic reaction + R-group
Phenol Beta-Glucosyltransferase (EC 2.4.1.35)
c
Generic reaction + stereochemistry
Glucose-1-Phosphatase (EC 3.1.3.10)
Partial chemistry
d
Partial reaction
Trypanothione Synthase (EC 6.3.1.9)
(i) + (ii)
(i)
+ ATP + Glutathione
Orthophosphate + ADP +
(ii)
+ ATP + Glutathione
Orthophosphate + ADP +
Different chemistry
e
Different types of reaction
Acetylenedicarboxylate decarboxylase (EC 4.1.1.78)
