# Supplementary material for: Characterising Complex Enzyme Reaction Data
Source: PLoS One. 2016 Feb 3;11(2):e0147952. doi: 10.1371/journal.pone.0147952 (PMC4740462; doi:10.1371/journal.pone.0147952)
Supplement: S1 Text — (PDF) [file pone.0147952.s007.pdf]

Supporting Information

Three additional forms of *partial* chemistry of the overall reaction

1. Two c*onsecutive* reactions and the *overall* reaction are all linked to the same EC number.
2. Two *consecutive* reactions are linked to the same EC number, the *overall* reaction is linked to a different EC number.
3. One *consecutive* reaction and the *overall* reaction are linked to the same EC number, the other *consecutive* reaction might be assigned to a different EC number or correspond to an uncatalysed reaction.

Testing the method to automatically label multi-reaction EC numbers according to the group of reaction diversity

The test set used to evaluate the performance of the method comprised fifty randomly-selected multi-reaction EC numbers, which were manageable by manual curation. We calculated true positives (TP), true negatives (TN), false positives (FP) and false negatives (FN) for each EC number in the test set and group of reaction diversity using manually-defined annotations as reference. The results are shown in Supplementary Figure 3.

Almost all the assignments made by the method for the fifty multi-reaction EC numbers were TPs or TNs, exceptions were two FPs for *different* types of reaction, six FNs for *generic* reaction + R-group and *partial* reaction and one EC number having an unbalanced reaction:

1. EC 1.1.1.222 was assigned to *different* reaction types due to an atom-atom mapping error in R03337 and R03339. This issue was addressed in the present development version of EC-BLAST.

2. EC 3.1.3.5 was assigned to *different* reaction types due to a data error, namely the different protonation state of the phosphate moiety in R02323 compared to the rest of reactions, leads EC-BLAST to detect a nonexistent formation of a O-H bond.

3. EC 1.2.1.4 was not assigned to g*eneric* reaction + R-group due to a data error concerning protonation states. Whereas R00634 presents a carboxylate group, R00711 and R05099 consider the corresponding chemical species as carboxylic acid.

4. EC 2.4.1.295 was not assigned to g*eneric* reaction + R-group due to difficulties in identifying R-groups. This is handled in development versions of the automatic method.

5. EC 3.5.1.2 was not assigned to g*eneric* reaction + R-group due to a data error concerning protonation states. Whereas R06134 presents a carboxylate group, R01579 and R00256 consider the corresponding chemical species as carboxylic acid.

6. EC 2.4.1.178 was not assigned to g*eneric* reaction + R-group due to similar difficulties in identifying R-groups as EC 2.4.1.295.

7. EC 1.1.1.42 was not assigned to *partial* reaction due to difficulties in detecting partial reactions. Here, R00268 and R01899 are *partial* reactions of the *overall* R00267 such that R00267 = R00268 + R01899.

8. EC 6.3.4.10 was not assigned to *partial* reaction due to similar difficulties in detecting partial reactions as in EC 1.1.1.42. Here, R01074 and R05145 are *partial* reactions of the *overall* R04582 such that R04582 = R01074 + R05145, considering C04763 = C06249 and C04727 = C06250.

9. EC 1.1.1.100 was not considered in the test due to a data error. R04534 is unbalanced because a proton is missing on the right-hand side of the reaction.
